# The Energetics of Streptococcal Enolase Octamer Formation: The Quantitative Contributions of the Last Eight Amino Acids at the Carboxy-Terminus

**DOI:** 10.1371/journal.pone.0135754

**Published:** 2015-08-19

**Authors:** Jack A. Kornblatt, Veronica Quiros, M. Judith Kornblatt

**Affiliations:** 1 Centre for Structural and Functional Genomics, Department of Biology, Concordia University, Montréal, Canada; 2 Department of Biology, Concordia University, Montréal, Canada; 3 Department of Chemistry and Biochemistry, Concordia University, Montréal, Canada; Nagoya University, JAPAN

## Abstract

The enolase produced by *Streptococcus pyogenes* is a homo-octamer whose overall shape resembles that of a donut. The octamer is best described as a tetramer of dimers. As such, it contains two types of interfaces. The first is common to almost all enolases as most enolases that have been studied are dimers. The second is unique to the octamers and includes residues near the carboxy-terminus. The primary sequence of the enolase contains 435 residues with an added 19 as an N-terminal hexahistine tag. We have systematically truncated the carboxy-terminus, individually removing the first 8 residues. This gave rise to a series of eight structures containing respectively, 435, 434, 433, 432, 431, 430, 429 and 427 residues. The truncations cause the protein to gradually dissociate from octamers to enzymatically inactive monomers with very small amounts of intermediate tetramers and dimers. We have evaluated the contributions of the missing residues to the monomer/octamer equilibrium using a combination of analytical ultracentrifugation and activity assays. For the dissociation reaction,

$$octamer\;\underset{}{⇐}\underset{}{\Rightarrow }\;8\;monomer$$

truncation of all eight C-terminal residues resulted in a diminution in the standard Gibbs energy of dissociation of about 59 kJ/mole of octamer relative to the full length protein. Considering that this change is spread over eight subunits, this translates to a change in standard Gibbs interaction energy of less than 8 kJ/mole of monomer distributed over the eight monomers. The resulting proteins, containing 434, 433, 432, 431, 430, 429 and 427 residues per monomer, showed intermediate free energies of dissociation. Finally, three other mutations were introduced into our reference protein to establish how they influenced the equilibrium. The main importance of this work is it shows that for homo-multimeric proteins a small change in the standard Gibbs interaction energy between subunits can have major physiological effects.

## Introduction

*Streptococcus pyogenes* is a known pathogen responsible for several diseases (see [1] for a review). The bacterium has a full complement of glycolytic enzymes and obtains much of its energetic requirements from glycolysis[2]. Interestingly, some of the glycolytic proteins are found not only intracellularly where they function in glycolysis but are also found on the surface of the bacterium[3,4]. Amongst those on the surface is Streptococcal enolase (Str enolase) which can, in an infected host, do two things: (1) bind a host’s plasminogen and (2) assist in the spread of infections [5–7]. It does the latter via its interaction with the plasminogen/plasmin system of the host.

**Fig 1:**
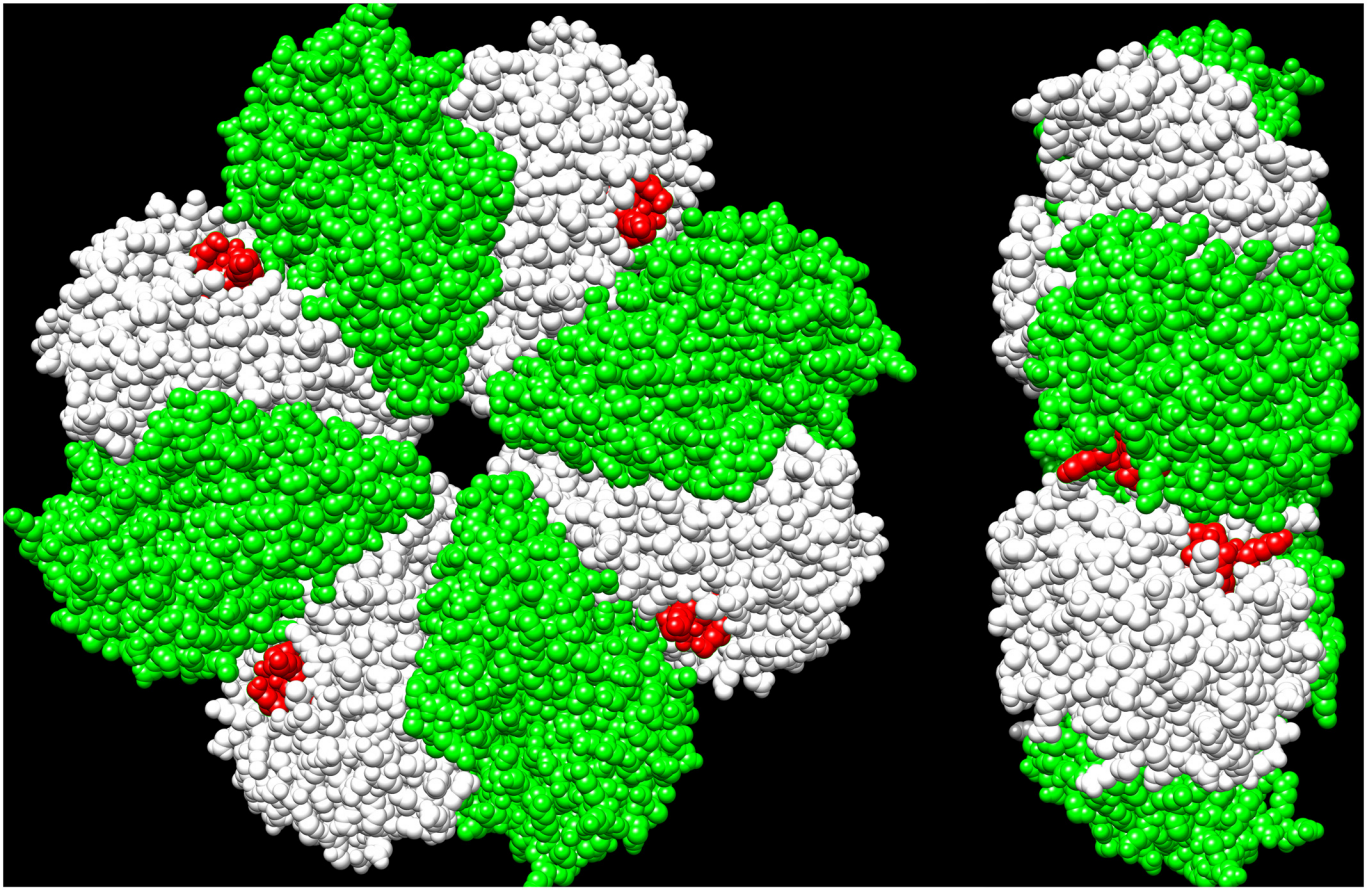
Str enolase (3ZLH.PDB) showing the alternating pattern of interfaces. The red represents residues 428–433 of the protein and indicates the position of the dimer-dimer interface; residues 434–435 do not appear in the X-ray structure and are not shown. The other interface is that of the monomer-monomer. Left side panel. Residues 428–433 appear on four subunits looking down on the donut and on the alternating subunits on the other side of the donut. Right side panel. Looking from the side of the donut clearly shows the nature of the dimer-dimer interface.

The native *Streptococcus pyogenes* enolase (E.C. 4.2.1.11) catalyzes the reversible interconversion of 2-phosphoglycerate and phosphoenolpyruvate. It is a homo-octamer (Fig 1) protein containing 435 amino acid residues in each monomer. As can be seen in the Fig, while all the subunits are identical, the arrangement of the subunits is such that the protein is a tetramer of dimers.

One of our laboratories (MJK) has been studying the dissociation/association reactions of different enolases, with a focus on the relationship between primary structure, quaternary structure and activity[8–14]. We asked the question: Can we destabilize the dimer-dimer interface and, if so, what is the net influence on the other properties of the protein? In this study we have focused on the removal of the carboxy-terminal amino acids, found at or close to the dimer-dimer interface. We found that their removal destabilizes the octameric structure and leads to both monomers and oligomers. The extent to which the native octamer/monomer equilibrium has been displaced by the introduction of voids in the place of amino acid residues has been quantified. The data reveal that removal of the individual amino acid residues destabilizes the dimer-dimer interface and that this in turn destabilizes the monomer/monomer interface. The energetic contribution of each of the last eight residues of each monomer to the overall octamer-monomer equilibrium is very small.

## Materials and Methods

The routine laboratory chemicals were ACS purity or higher. 2-phosphoglyceric acid was synthesized and purified as described[15]. The buffer used throughout the study was TME-SO_4_. It consisted of 50 mM Tris, 1 mM MgSO_4_, 0.1 mM EDTA, 44 mN H_2_SO_4_, pH 7.4. The buffer used during enzymatic assays was 50 mM HEPES, 1 mM MgSO_4_, 1 mM 2-phosphoglyceric acid, pH 7.4.

The reference protein was the enolase from *Streptococcus pyogenes* F137L/E363G (abbreviated: Str enolase 137/363). It differs from the sequence in the protein data bank (accession number NP_268959.1, abbreviated here: Str enolase DB) at the two indicated positions. All aspects of dealing with the DNA clone, plasmid maintenance, site directed mutagenesis, growth of the bacteria and expression and purification of the proteins have been described[8]; *E*. *coli* containing the truncations from -2 to -8 were grown at 18°C instead of 37°C. The oligonucleotides used to construct the truncations were as follows:

Str enolase 137/363–1 (ΔK435)

5’- CAA ATC ATT CTA TAA CTT AAA ATA GTA GTA GGA TCC GGC TGC -3’

5’- GCA GCC GGA TCC TAC TAC TAT TTT AAG TTA TAG AAT GAT TTG -3’

Str enolase 137/363–2 (ΔK434-K435)

5’- CAA ATC ATT CTA TAA CTT ATA GAA ATA GTA GGA TCC GGC -3’

5’- GCC GGA TCC TAC TAT TTC TAT AAG TTA TAG AAT GAT TTG -3’

Str enolase 137/363–3 (ΔL433-K435)

5’- GTA TCA AAT CAT TCT ATA ACT AGA AAA AAT AGT AGG ATC CG -3’

5’- CGG ATC CTA CTA TTT TTT CTA GTT ATA GAA TGA TTT GAT AC -3’

Str enolase 137/363–4 (ΔN432-K435)

5’- GTA TCA AAT CAT TCT ACT AGT TAA AAA AAT AGT AGG ATC CG -3’

5’- CGG ATC CTA CTA TTT TTT TAA CTA GTA GAA TGA TTT GAT AC -3’

Str enolase 137/363–5 (ΔY431-K435)

5’- CAA AGG TAT CAA ATC ATT CTA GAA CTT AAA AAA ATA GTA GG -3’

5’- CCT ACT ATT TTT TTA AGT TCT AGA ATG ATT TGA TAC CTT TG -3’

Str enolase 137/363–6 (ΔF430-K435)

5’- CAA AGG TAT CAA ATC CTA GGA TAA CTT AAA AAA ATA GTA GG -3’

5’- CCT ACT ATT TTT TTA AGT TAT CCT AGG ATT TGA TAC CTT TG -3’

Str enolase 137/363–8 (ΔK428-K435)

5’- TTT TAA GTT ATA GAA TAG CTA GAT ACC TTT GTA TTG AGC AAC -3’

5’- GTT GCT CAA TAC AAA GGT ATC TAG CTA TTC TAT AAC TTA AAA -3’

Sequencing of the DNA for each truncation confirmed that the truncations occurred at the correct position. The molecular masses of all eight purified proteins were verified by mass spectroscopy. The purified proteins were stored in saturated ammonium sulfate until used. Three days prior to an experiment, an aliquot of the ammonium sulfate suspension was centrifuged, the pellets dissolved in TME-SO_4_ and dialyzed three times against the same buffer. The resulting solutions were centrifuged at 13, 000 RPM for five minutes. Each dialyzed protein was shown to consist of a single band on SDS-PAGE. Protein concentrations were estimated at 280 nm using a molar extinction coefficient of 43, 300 M^-1^cm^-1^.

**Fig 2:**
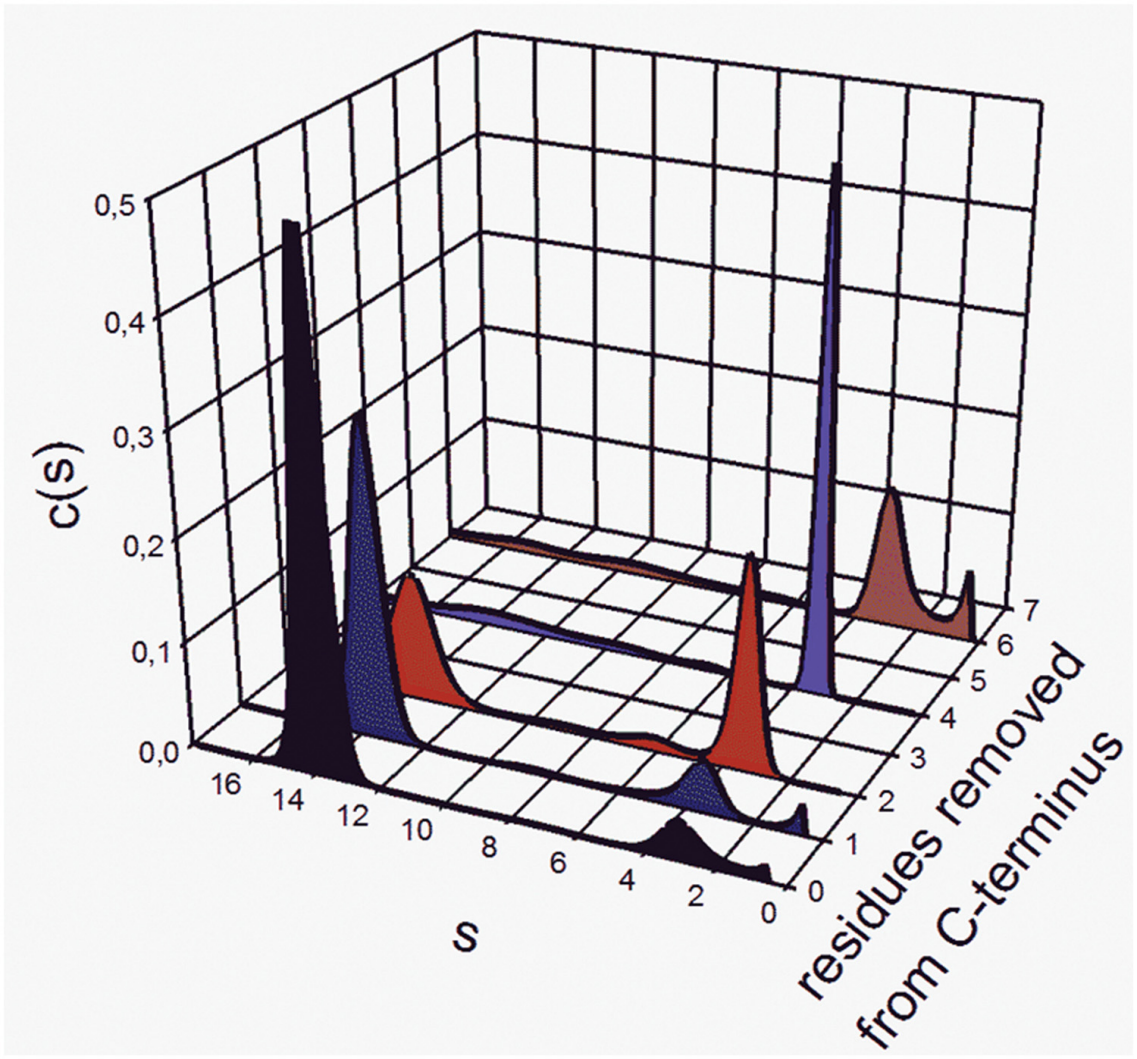
The truncation of Str enolase 137/363 from the carboxy-terminus results in dissociation of the octamer and the concomitant formation of monomers. The reference sample (black curve) was Str enolase 137/363. All samples were prepared in TME-SO_4_. In the truncated samples, the data at s > 18 are not shown. These are aggregates and do not contribute to the calculation of either Kd or ΔG°. Since there are more aggregates in the -4, -6 and -8 truncations than in the -2, the monomer peak in the -2 sample is larger than in the other truncations. The samples chosen for the five curves were chosen because they corresponded to the average values shown in Table 1.

Analytical ultracentrifugation (AUC) was performed on a Beckman XL-I thermostated at 20°C. Speeds were controlled between 30 000 RPM and 37 000 RPM depending on presence of aggregated species. Monomers and dimers were easily separated at 37 000 RPM but it was necessary to use lower speeds to clearly identify other species. The detailed use of this centrifuge has been described[16]. Data were evaluated using Sedfit [17,18]. We used c(s) to estimate the distribution of monomers and octamers. The equilibrium between the two species is thought to be particularly slow such that the equilibrium species can be reliably evaluated. When we centrifuged the same samples on repeated days, the percent of the two species remained constant within experimental error. In order to accurately estimate the percentage of monomers and octamers, independent samples were run a minimum of three times (Str enolase DB and Str enolase 137/363–8) and a maximum of 13 times. One sample of Str enolase 137/363 was included with every run of truncated Str enolase. The percentages of monomer and octamer were estimated from the size distribution plot in Sedfit c(s) and converted into molar concentrations by referencing the loading concentration. This worked well for the series Str enolase 137/363 to Str enolase 137/363–5 but the Str enolase 137/363–6 and -8 concentrations of octamer had to be estimated from activity measurements since the octamer fraction of Str enolase 137/363–6 and -8 in the AUC was so low as to be not measurable. Using activity assays, we could estimate the total amount of octamer as 0.0002% of the concentration of the Str enolase DB. As can be seen in Fig 2, the concentration of monomer for the Str enolase -6 could easily be estimated from the AUC determinations. The same is true for Str enolase 137/363–8 (data not shown).

Activity measurements were performed as previously described [8] by following the formation of phosphoenolpyruvate from 2-phosphoglycerate at 20°C at 240 nm (extinction coefficient 1330 M^-1^cm^-1^). The assays were repeated eight times on independent samples in order to have reasonable precision for calculating the concentration of octamer in the stock solution. The percentage octamer determined in the AUC paralleled the turnover numbers in the first six samples, Str enolase 137/363, -1, -2, -3, -4, -5. We felt justified therefore in assuming that the residual activity in the -6 and -8 samples represented the amount of octamer present. While in this study the activity measurements scaled uniquely with the total amount of octamer present, in other work involving Str enolase dissociated by treatment with the chaotrope, NaClO_4_, activity correlated with the sum of non-monomeric species[8].

Data from the analytical ultracentrifuge were evaluated for the percent of the loading concentration present as monomers or octamers of Str enolase. These values were used to calculate the standard Gibbs energy of dissociation for the dissociation reaction. It is important to recognize that the following calculations make no assumptions about how the truncations influence the octamer/monomer equilibrium.

$$octamer\;\underset{}{⇐}\underset{}{\Rightarrow }\;8\;monomer$$

$$K_{d}\;=\;{\left[monomer\right]}^{8}/\left[octamer\right]$$

Where

[monomer] =molar loading concentration in the AUC run*% monomer

and

[octamer]=molar loading concentration in AUC run*% octamer

and

$$\Delta G^{{}^{\circ}}\;=\;-RTlnK_{d}$$

Activity measurements were also used to calculate the free energies of dissociation of the octameric enolase. In this case there is a hidden assumption: The octamer, truncated or full length, is folded such that the active site is correctly folded.

$$\left[octamer\right]=\;\frac{\left(turnover\;number\;of\;Str\;enolase\;species\right)}{\left(turnover\;number\;of\;Str\;enolase\;DB\right)*concentration\;of\;species\;in\;AUC)}$$

The AUC data indicate that the Str enolase DB, and the Str enolase DB E363G are “virtually” fully octameric. There is a measurable amount of mass in the monomer region of both DB and DB E363G but it is minuscule relative to all other forms of the Str enolase. Therefore we assume that all loss of activity is the result of loss of the Str enolase octamer.

[monomer] =molar loading concentration in the AUC run*% monomer

The calculations of Kd and ΔG° were as above.

Circular dichroic measurements were carried out with Jasco 810 and 815 spectropolarimeters thermostated at 20°C. Data were collected between 185 nm and 280 nm. The bandwidth was 1 nm, pitch was 0.2 nm; five spectra were accumulated and averaged using a cuvet with a path length of 0.01 cm. The TME-SO_4_ buffer was well suited to the measurements; the high voltage never exceeded 550 volts. Data were submitted to the Birbeck College site, Dichroweb, and evaluated using the methods outline by Whitmore and Wallace (CDSSTR, data base 6 [19,20]).

## Results

The genome of *S*. *pyogenes* was originally sequenced by Ferretti et al.[21]. Str enolase was subsequently cloned by the group of Vijay Pancholi who generously provided us with the clone. The sequence of this protein is in the NCBI data bank and we refer to it as Str enolase DB (Accession number NP_268959.1). When we sequenced the provided clone of DNA we found that two mutations had inadvertently arisen[8]. The changes are F137L and E363G; we refer to the resulting protein as Str enolase 137/363. We have chosen to work with this form of the protein since it is partially dissociated. This makes it easier to examine effects of other mutations on the equilibrium. The X-ray crystal structure of Str enolase has recently been deposited in the data bank (3ZLH.PDB Fig 1). The coordinates were kindly given to us by the authors (Cork, Ericsson, Law, Casey, Valkov, Bertozzi, Stamp, Aquilina, Whisstock, Walker and Kobe) [22]. This protein contains one mutation (E77K) relative to the Str enolase DB. Importantly, the two C-terminal lysines do not appear in the crystal structure, nor in the structure (1W6T.PDB) of the enolase from *S*.*pneumoniae*. Str enolase 137/363 is the reference structure for all the work reported here.

The calculations made in this report are based on AUC and activity measurements. The thermodynamic results have been summarized and are shown in Table 1. The quality of the AUC data can be seen in Fig 2 while the activity data are summarized in Table 2.

**Table 1:** Thermodynamic parameters of the mutated and truncated forms of Str enolase.

Protein Form	Number individual AUC runs	ΔG° ± (standard error) of dissociation (average) based on AUC determinations (kJ)	ΔG° of dissociation (average) based on eight activity determinations (kJ)
Str enolase 137/363	13	247 ± 11	235 ± 3
Str enolase 137/363–1	8	235 ± 5	234 ± 2
Str enolase 137/363–2	9	211 ± 8	198 ± 2
Str enolase 137/363–3	8	214 ± 11	192 ± 1
Str enolase 137/363–4	6	187 ± 1	188 ± 1
Str enolase 137/363–5	6	197 ± 0	199 ± 3
Str enolase 137/363–6	3	210 Unreliable	166 ± 2
Str enolase 137/363–8	3	230 Unreliable	178 ± 2
Str enolase DB	3	306 ± 7	304 ± 4
Str enolase DB F137L	7	264 ± 2	261 ± 4
Str enolase DB E363F	6	306 ± 3	310 ± 4

**Table 2:** Activity and CD characteristics of mutated and truncated forms of Str enolase.

Protein Form	Molecular Activity (min^-1^) ± Std error	% Helix	% Strand
Str enolase 137/363	5249 ± 65	47	18
Str enolase 137/363–1	4045 ± 40	47	16.5
Str enolase 137/363–2	1727 ± 24	33	22
Str enolase 137/363–3	497 ± 3.6	28	24
Str enolase 137/363–4	27 ± 2.7*	20	25
Str enolase 137/363–5	0.36 ± 0.03*	22	26
Str enolase 137/363–6	0.22 ± 0.06*	17	29
Str enolase 137/363–8	0.08±0.01*	ND	ND
Str enolase DB	8173 ± 47	52–55	8–14
Str enolase F137L	7155 ± 22	51	13
Str enolase E363G	8460 ± 96	54	13

ND = not determined,

* The standard errors as a percentage of the activity for these samples are high because the concentration of protein in the assays is very high and there is reactivation of the enolase during the assay.

**Fig 3:**
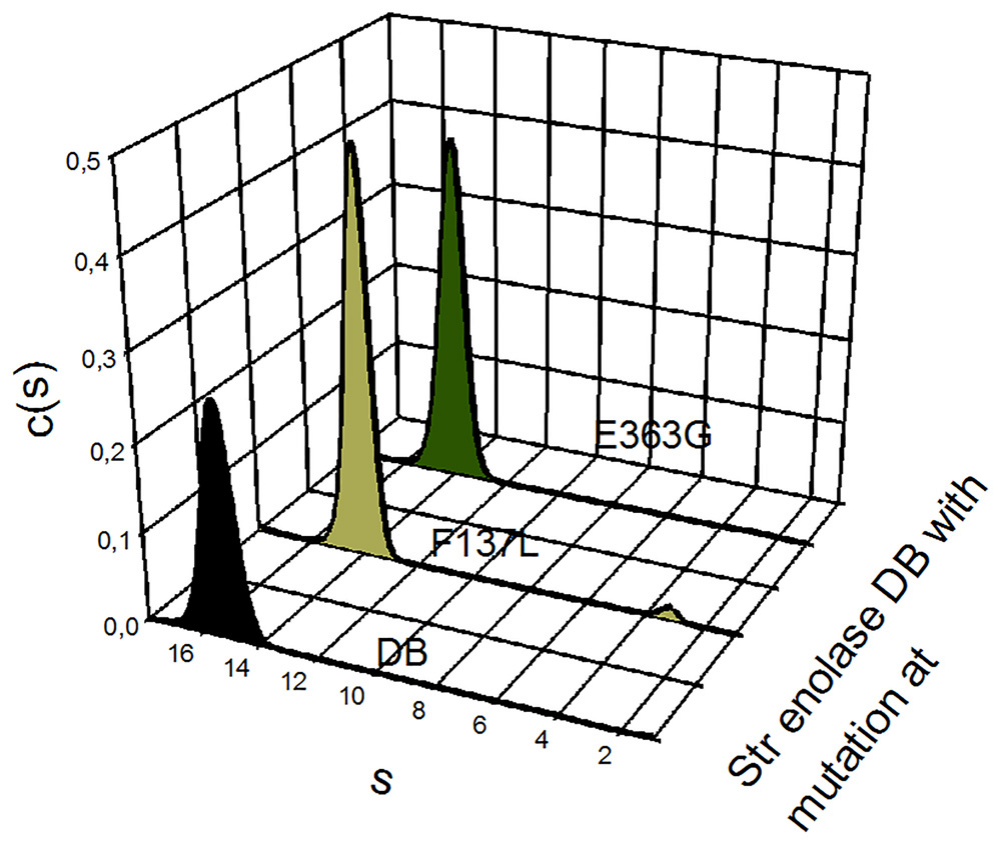
The influence of the individual mutations F137L and E363G on the Str enolase DB as determined by AUC. The reference sample (black curve) was Str enolase DB, F137L is light green and E363G is dark green. All samples were prepared in TME-SO_4_. The feature which should be noted is the small peak at s = 3.5 in the F137L c(s) vs s plot which is present to a much smaller degree in the other two plots.

The extent to which the two measurements of ΔG° agree (Table 1), provides us with some confidence that the two techniques are monitoring the same phenomenon. It is worth noting that removal of residue 435 results in little change in the octamer/monomer equilibrium but that removal of residues 434 and 435 causes a loss of ca 20 kJ in stability. This is somewhat curious since neither residue appears in the X-ray structure and is therefore probably quite unstructured. It is also interesting to note that truncation at residue 433 (Str enolase -3) appears to have little influence on the overall octamer/monomer equilibrium; the same is true for residue 431 (Str enolase -5) which may contribute to stabilizing the octamer. The loss of stability on removal of residues 1 through 6 relative to 1 through 5 is probably significant but our ability to accurately measure the concentration of octamer in the AUC (see Fig 2) or activity (Table 2) for the Str enolase 137/363–6 is poor. In order to obtain the activity estimates of the -6 truncation, it required 6 nmol of monomer in the 0.5 mL assay; this contributes an absorbance of close to one at 240 nm. The precision of the very low activity proteins is high but the accuracy is questionable. The major loss in stability is found in comparing the Str enolase DB and the Str enolase F137L. Representative AUC scans for the Str enolase DB derived mutations are shown in Fig 3 and the statistically significant calculated data in Tables 1 and 2. The feature to note in Fig 3 is the small low s value peak at s = 3.5. This peak represents the small amount of dissociation from octamers to monomers but also the large loss of stability resulting from the F137L mutation. We are currently trying to determine whether the loss in stability is the result of steric factors or pi-charge interactions [23] that result from phenylalanine 137 interacting with nearby charged residues. It has been pointed out that at any given temperature the overall structure of a protein is the small algebraic sum of a large number of contributions, some large and others not so large[24]. Since sequestering an amino acid residue into the protein structure must necessarily involve a negative ΔS, (-TΔS must therefore be positive) the very small change in ΔG° could easily be the result of both entropic and enthalpic factors.

Also interesting is the comparison between the free energies of the equilibrium between the Str enolase DB, the two Str enolase mutant forms (F137L and E363G) and the Str enolase 137/363. Str enolase DB and Str enolase E363G are clearly the thermodynamically most stable structures analyzed here. The single mutation F137L accounts for most of the destabilizing influence relative to the comparison between Str enolase DB and Str enolase 137/363.

We have also determined the influence of the truncations on the CD spectra of the Str enolase (Table 2). Str enolase DB contains the highest percentage of helix and the lowest percentage of strands. This is followed by a gradual diminution of the percentage of helix as one truncates more residues from the carboxy-terminus. The percentage of strand increases as the percentage helix goes down and the percentage unordered also increases. Since we start to see very high molecular mass aggregates when we truncate more than three residues, the meaning of the CD spectra of the higher truncations is not clear. In the latter cases, the loss of helix is due to multiple species present and not just monomers and octamers.

## Discussion

Constructing specific truncated proteins is not a new idea. Starting in the late 1960s and using solid support synthesis Merrifield showed that truncated enzymes could be active and that in some cases the inactive truncated form could be activated by mixing with specific peptides [25–30]. More recently, Schwartz and Merz [31] have shown that they could trap intermediates in the process by which membrane vesicles fuse using truncated SNARES. In most cases the goal of the work was to determine whether a given residue, group of residues or a loop was a requirement for activity, for a given protein interaction with another protein or assembly of a complex. There has been little emphasis on just how much a given residue contributes to the overall energetics of assembly. That was the principle goal of this study. How much energy do the individual residues of Streptococcal enolase contribute to the formation of the octameric final structure? The answer that has emerged is clear. Starting from the sequence found in the Protein Data Bank (Accession number NP_268959.1), changing one residue F137 to leucine reduces the stability of the octamer by 5 kJ/mol/monomer; in other words, the change is comparable to removing one rather weak hydrogen bond (Maurice Huggins (ca 1920) and Latimer and Rodebush (1922) cited by Martin and Derewenda [32]). The truncations destabilize the octamer by less than five kJ/mol/monomer for each residue removed. Since the octamer contains eight subunits, the change in the position of the monomer/octamer equilibrium is significant as can be seen in Fig 2.

There has been a significant amount of work done on mutant forms of enolase from *Streptococcus pyogenes* and *Streptococcus pneumoniae* [7,8,22,33–35]. Cork et al. [22] recently found that changing K362 to alanine destabilizes the Str enolase. Much of the rest of the above cited work was aimed at determining the role of specific residues, especially residues 434 and 435, in the binding of plasminogen. It is clear from the work presented in this manuscript that removal of those two residues has significant effects on activity, secondary and quaternary structure. Without a thorough characterization of the mutant forms, it is not possible to evaluate the contribution of specific residues to the binding of plasminogen. When one considers the more general question of what is it that determines whether a protein monomer polymerizes to form a stable dimer, tetramer or octamer, the answer must involve not only the residues at the prospective interface but also the geometry that allows the interfaces to approach one another. In the case of the octameric enolase, the two types of interface are not unusual. The monomer-monomer interface has a ratio of charged/hydrophobic residues of about 1.1 whereas the comparable ratio for the dimer-dimer interface is about 0.8 [34]. The monomer-monomer interface is rather planar. Interfacial residues are the purview of the amino acids themselves and the geometry they can assume. Internal residues, in turn, determine whether the overall aspect of the interface is correctly positioned so as to allow a given oligomer to form [36,37].

**Fig 4:**
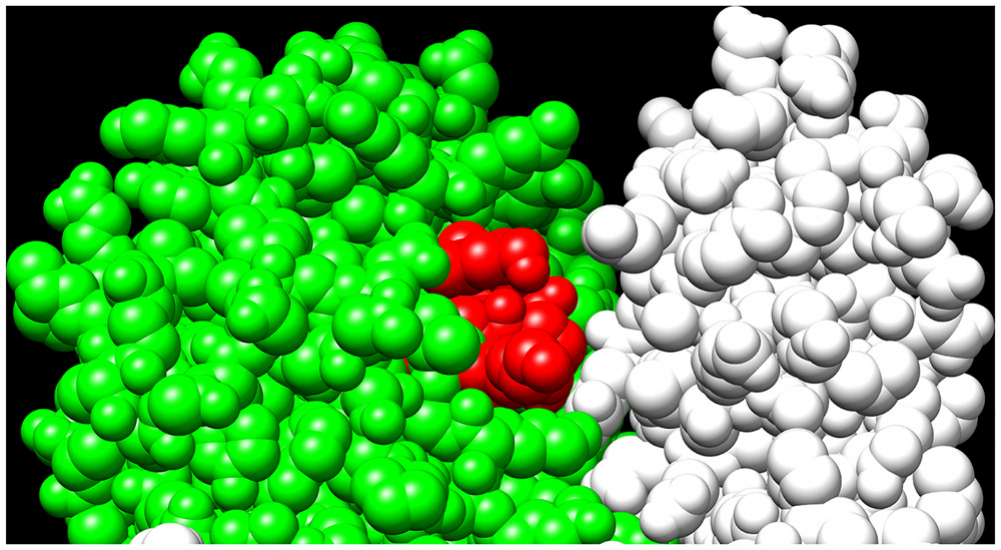
The hole which would be created but is here covered by the eight (red) c-terminal amino acid residues from Str enolase 137/363. Of the visible residues removed, three are polar, one is hydrophobic and one is mixed. The two terminal lysines are not visible but are clearly polar.

It has repeatedly been stated that nature abhors a vacuum. The aphorism may be true in the macroscopic world but not necessarily in the microscopic. If the energetic costs of introducing or maintaining a vacuum, a large unoccupied hole, are great enough, the hole gets filled with water [38–42]. It can be filled by compression of nearby residues caused by hydrostatic pressure [40,43–49]. Fig 4 shows a close up of the region containing the eight residues (428–435) that have been removed. Since the crystal structure does not show the two terminal residues, only 428–433 are shown. The hole, which would be left by the removal of the red residues (KSFYNLKK), is extensive, mostly composed of polar residues, and would account for approximately 995 Å^3^ (calculated with PCModel v. 6.0) or 850 Å^3^ (calculated with UCSF Chimera 1.10). Considering that a water molecule occupies about 25 Å^3^ the hole is large enough to accommodate a significant number of waters. Hydration of the eight pockets of the octamer is undoubtedly a major contributor to the destabilization of the octamer and undoubtedly contributes in a major way to the large loss of activity and quaternary structure reported here. The creation of holes may also result in small rearrangements of the residues at the interface, thereby weakening the cohesive forces. The removal of residues also exposes hydrophobic surfaces; small rearrangements might take place in order to shield these surfaces from water. Since the changes in the standard Gibbs interaction energy per individual residue are so very small it is not likely the answer to the question of a proximate cause will be forthcoming any time soon. After all, decreasing the length of a single H·····N interaction changes the van der Waals potential by about 0.5 kJmol^-1^ [50]. If one multiplies that number by eight, one begins to have a feel for the difficulty in establishing proximal causes for multimers in general and octamers in particular.

## Supporting Information

S1 Table AUC measurements and calculations. (XLSX)

S2 Table Activity measurements and calculations. (XLSX)

S1 File All original CD files. (ZIP)
